# The Effects of Serious Games on Cardiopulmonary Resuscitation Training and Education: Systematic Review With Meta-Analysis of Randomized Controlled Trials

**DOI:** 10.2196/52990

**Published:** 2024-02-06

**Authors:** Pengfei Cheng, Yangxi Huang, Pengyu Yang, Haizhen Wang, Baichao Xu, Chaoran Qu, Hua Zhang

**Affiliations:** 1 Department of Nursing The Second Affiliated Hospital of Zhejiang University School of Medicine Hangzhou China; 2 School of Nursing The University of Hong Kong Hong Kong China; 3 Department of Nursing West China Hospital, Sichuan University Chengdu China; 4 Department of Physical Education Hainan Medical University Haikou China; 5 Department of the Operating Room Shenzhen People’s Hospital (The Second Clinical Medical College, Jinan University; The First Affiliated Hospital, Southern University of Science and Technology) Shenzhen China; 6 International Nursing School Hainan Medical University Haikou China; 7 Key Laboratory of Emergency and Trauma Ministry of Education Haikou China

**Keywords:** CPR, education, meta-analysis, serious game, training

## Abstract

**Background:**

Serious games have emerged as an innovative educational strategy with the potential to significantly enhance the quality and effectiveness of cardiopulmonary resuscitation (CPR) training. Despite their promise, there remains a degree of controversy when comparing the advantages of serious games with traditional CPR training methods. This study seeks to provide a comprehensive assessment of the impact of serious games on CPR training and education by systematically analyzing the results of previous research.

**Objective:**

This study aimed to assess the effect of serious games on CPR training and education by summarizing and pooling the results of previous studies.

**Methods:**

We conducted a thorough and systematic search across 9 prominent web-based databases, encompassing the period from the inception of these databases until April 1, 2023. The databases included in our search were PubMed, Cochrane Library, Wiley Online Library, EBSCO (PsycInfo), SpringerLink, Chinese Biology Medicine Disc, Vip Journal Integration Platform, Wanfang Database, and Chinese National Knowledge Infrastructure. The studies selected adhered to the following criteria: (1) being a randomized controlled trial comparing serious games and traditional methods for CPR training; (2) having participants aged 12 years or older in CPR; (3) having an experimental group using serious games and a control group using nongame methods for CPR instruction; and (4) having outcomes including theoretical and skill assessments, compression depth, and rate. The Cochrane risk of bias assessment tool was used to evaluate the risk of bias. Data analysis was performed using RevMan (version 5.3; Cochrane Training), and mean differences (MDs) and standardized mean differences (SMDs) with 95% CIs were used to calculate continuous variables.

**Results:**

A total of 9 articles were included, involving 791 study participants, of whom 395 in the experimental group taught CPR training using serious games and 396 in the control group taught CPR training using traditional methods. The results of our meta-analysis indicate that the use of serious games in CPR training yields outcomes that are comparable in effectiveness to traditional training methods across several key areas. Specifically, serious games demonstrated equivalence to traditional formats in theory assessment (SMD –0.22, 95% CI – 0.96 to 0.51; *P*=.55), skill assessment (SMD –0.49, 95% CI –1.52 to 0.55; *P*=.36), compression depth (MD –3.17, 95% CI –0.18 to 6.53; *P*=.06), and compression rate (MD –0.20, 95% CI –7.29 to 6.89; *P*=.96).

**Conclusions:**

In summary, serious games offer a viable and effective CPR education approach, yielding results comparable to traditional formats. This modality is a valuable addition to CPR training methodologies. However, caution is warranted in interpreting these findings due to limited controlled trials, small sample sizes, and low-quality meta-analyzed evidence.

## Introduction

### Background

Out-of-hospital cardiac arrest (OHCA) is a critical medical emergency characterized by the sudden cessation of heart function, resulting in an abrupt loss of blood flow. OHCA incidents frequently occur in community settings, schools, homes, and public places [1]. Despite sustained efforts, OHCA survival rates remain disheartening, largely due to modifiable factors such as bystander cardiopulmonary resuscitation (CPR), automated external defibrillator (AED) use, and the timing of emergency medical services (EMS) intervention [2,3]. In the United States, OHCA affects over 88.8 adults per 100,000 adults annually, with a mere 9.0% discharge survival rate, as reported by the American Heart Association [4]. Similarly, in Europe, the annual incidence of OHCA among adults ranges from 67 to 170 per 100,000, with discharge survival rates varying from 0% to 18% [5]. In China, more than 540,000 individuals experience OHCA each year, but the survival rate remains at approximately 2% [6]. These statistics underscore that OHCA, despite regional disparities, has emerged as a substantial public health challenge, imperiling the well-being of citizens [7]. OHCA is typified by sudden respiratory distress, pulse cessation, and loss of consciousness, necessitating immediate and effective first-aid measures within the critical 4-minute window [8]. However, current prehospital EMS services often struggle to reach the scene promptly to address emergencies in public spaces [9]. Consequently, first responders (FRs), nonmedical professionals in public areas, shoulder the responsibility of on-site rescue efforts [10]. Swift and efficient basic life support interventions administered by FRs not only create a vital time buffer for EMS teams to arrive but also substantially elevate the chances of patients with OHCA surviving [11].

CPR, encompassing artificial respiration and chest compressions, stands as one of the simplest and most universally applicable techniques for basic life support during OHCA emergencies [12]. The quality of chest compressions holds immense significance in preserving organ perfusion. Consequently, the timely and effective administration of CPR plays a pivotal role in determining both the survival rate and neurological outcomes for patients with OHCA [3]. To enhance the widespread adoption of CPR and ensure that more individuals are proficient in this vital first-aid technique, the World Health Organization and the International Liaison Committee on Resuscitation endorsed the “Kids Save Lives” statement, which calls for CPR training for students, adolescents, and adults aged 12 years or older who already have the physical fitness and learning ability to understand and remember CPR skills to empower young people, including children aged 12 years, with CPR skills. Develop a generation of proactive and empowered community members who are expected to make a difference in emergency situations, especially in the context of OHCA, with the goal of increasing survival and improving long-term outcomes for patients with OHCA [13,14].

Serious games are increasingly used in medical education, encompassing medical theory instruction, clinical skills training, cognitive rehabilitation exercises, and patient health education. The integration of serious games into medical simulation programs is seen as a means to enhance the efficiency and effectiveness of training programs [15,16]. Otero-Agra et al [17] used serious games to instruct middle school students in CPR, revealing that 61.7% of participants acquired correct CPR techniques, with 93.4% achieving an average chest compression depth exceeding 50 mm. These results endorse serious games as effective tools for knowledge acquisition and the mastery of high-quality CPR skills. To optimize their use as an educational strategy, serious games must possess robust content and cater to the target audience. Integrating learning theory with game requirements enhances student engagement and ensures the efficacy of learning [18]. High fidelity is crucial, especially for medical students, as the knowledge and skills acquired in serious games will be applied in future clinical practice involving real patients. High-fidelity serious games bridge the gap between virtual gaming scenarios and clinical reality, boosting rescue confidence and self-efficacy [19]. Creutzfeldt et al [20] used serious games based on massively multiplayer virtual worlds technology to train 36 high school students in CPR. After 90-120 minutes of game-based sessions, participants reported a significant increase in self-efficacy, endorsing the effectiveness of serious games for CPR instruction. Moreover, serious games can incorporate adaptive learning features, adjusting difficulty and content based on the learner’s proficiency, ensuring tailored learning for individuals with varying CPR skill levels [21].

The incorporation of serious games into CPR training aims to enhance the learning process by rendering it more engaging, interactive, and effective. Compared to conventional methods relying on lectures, videos, and hands-on practice, serious games make the learning experience more enjoyable, interactive, and motivation-driven, integrating features such as scores, levels, and rewards [21,22]. Notably, serious games for CPR training are user-facing, offering immediate training opportunities, flexible learning schedules, and detailed real-time feedback on CPR performance [23]. In contrast, traditional teaching models often limit training opportunities, providing delayed feedback, particularly in large-scale group activities where individual feedback is frequently overlooked [24]. A systematic review by Lim et al [25] underlines that the absence of regular retraining and effective feedback in traditional CPR education can impact skill retention. Serious games address these shortcomings by providing continuous opportunities for practice and feedback. Moreover, serious games support collaborative learning, enabling learners to respond jointly to virtual CPR scenarios and develop teamwork and communication skills. They also offer diverse immersive first aid scenarios with varying causes of cardiac arrest, an aspect unattainable in traditional teaching formats [16,26]. This multifaceted approach not only compensates for the deficiencies in traditional methods but also promotes a dynamic and engaging learning environment in CPR training. Considering the advantages mentioned above, the 2020 American Heart Association Guidelines for Cardiopulmonary Resuscitation and Emergency Cardiovascular Care recommended the incorporation of serious games into CPR training and education to enhance teaching methods and improve instructional quality, taking into account advancements in training equipment and teaching formats [27]. However, Dankbaar [28] concluded that serious games have limitations in terms of time and their ability to provide learners with sufficient knowledge acquisition and complex skill improvement. In summary, there exists a degree of controversy regarding the impact of serious games on CPR training and education. Therefore, we aimed to conduct a meta-analysis to determine the effectiveness of serious games in CPR training and education.

### Research Gap and Aim

While numerous researchers have explored and experimented with serious games for CPR training, published randomized controlled trial (RCT) studies have explored and experimented with the effect of serious games applied to CPR training, and their effectiveness has been proven and supported [16,29]. However, due to the limitations of research, the generalization of research conclusions is affected. Specifically, (1) these RCTs were single-center studies with small sample sizes; (2) specific serious games limit the reliability of the findings in different settings of serious games or target populations; (3) outcomes were mostly assessed by questionnaires, and there were a lack of reliable, automated, and repeatable methods to measure their efficacy; and (4) there is a lack of methodological specifications and standard protocols for the use of serious games. Furthermore, there is a lack of systematic evaluation or meta-analysis of the effectiveness of serious games-based CPR training. Consequently, it is necessary to quantitatively analyze the objective effect of serious games–based training through meta-analysis. In view of this, we conducted a meta-analysis to comprehensively evaluate the effect of serious games on CPR training and teaching.

## Methods

### Overview and Registration

This systematic review adheres to the guidelines set forth by the PRISMA (Preferred Reporting Items for Systematic Reviews and Meta-Analyses) [30] and was registered in advance in the PROSPERO (International Prospective Register of Systematic Reviews) database (registration number CRD42023423089).

### Search Strategy

Our search was conducted in several databases, including PubMed, Cochrane Library, Wiley Online Library, EBSCO (PsycInfo), and SpringerLink. Besides, Chinese databases, including the China National Knowledge Infrastructure (CNKI), China Biomedical Literature Database, VIP Journal Integration Platform, and Wanfang Database, were searched. The search was conducted from the inception of the databases until April 1, 2023. We limited the publication language to English and Chinese. English search terms included “serious game,” “gam*,” “cardiopulmonary resuscitation,” “CPR,” “basic life support,” “BLS,” “first aid training,” “resuscitation education,” “emergency skill,” etc. The search involved a combination of subject terms and free words, with a manual retrospective search of references and associated literature to ensure a comprehensive search of relevant studies. Multimedia Appendix 1 provides detailed information on the search strategies, including search terms, and the process used.

### Eligibility Criteria for This Review

The eligibility of studies was assessed based on the following criteria: (1) the study type should be an RCT comparing the effectiveness of serious games with other traditional training methods for teaching CPR; (2) the study population should include participants aged 12 years or older who participated in CPR training or first aid training that covered basic life support for CPR; (3) interventions in the experimental group should involve the use of serious games for CPR training instruction, while the control group should receive other methods of CPR theory and skills training instruction excluding serious games, with no limitations on the types of games or software used; and (4) outcome measures should include one or more of the indicators of theoretical assessment, CPR skill assessment, compression depth, and compression rate. Additionally, duplicate or multiple manuscripts, literature in languages other than Chinese or English, literature with inaccessible full text, incomplete or missing data, improper data collection, or errors in statistical methods were excluded.

### Screening Process

Two authors (PC and PY), who were trained in evidence-based methods, independently conducted the screening of literature and extraction of data. All references were managed using EndNote X9 (Clarivate), a reference management software. After removing duplicates, the remaining references were first screened based on titles and abstracts. Subsequently, full-text screening was performed independently by the authors in duplicate to determine the inclusion of literature. Disagreements were resolved through discussion or adjudication by a third author (HZ).

### Quality Assessment

The Cochrane handbook’s criteria for assessing the risk of bias in RCTs were used to evaluate the methodological quality of the trials [31]. The assessment covered various aspects, including selection bias, concealment of the allocation scheme, implementation bias, measurement bias, missed visit bias, reporting bias, and other biases. Each item was categorized as “low risk of bias,” “unclear,” or “high risk of bias.” In cases where differing opinions arose, a third author (HZ) was involved to reach a consensus.

### Data Extraction

For data extraction, we used Excel (2010; Microsoft Corporation) to create a standardized form. The form included the following information: (1) basic details such as the first author, publication year, and country of the study; (2) population characteristics, sample size, and information about the serious games used in training and teaching; (3) specific interventions for the test and control groups; and (4) outcome measures and the tools used for measurement.

### Statistical Analysis

Data analysis was carried out using RevMan (version 5.3; Cochrane Training). To assess heterogeneity, the Q test and the *I*^2^ test were used. If the resulting *P* value was greater than or equal to .1 and *I*^2^ was less than or equal to 50%, it indicated low heterogeneity among the findings, leading to the selection of the fixed-effects model for meta-analysis. Otherwise, the random-effects model was used. When comparing groups, continuous variables were analyzed using mean difference (MD) if the same measurement instrument was used or standardized mean difference (SMD) if different instruments were used. Both effect measures were reported with 95% CIs. For continuous data that did not follow a normal distribution in the included studies and were expressed as medians, extreme values, or quartiles, a specific web-based formula calculator developed by Professor Luo et al [32] from Hong Kong Baptist University was used. This calculator, designed for meta-analysis data conversion, enabled statistical estimation of the data. Leave-one-out analysis was used to conduct sensitivity analysis, that is, omitting one study at a time from the meta-analysis and examining the impact on the overall effect size, then judging the robustness and reliability of the results and exploring the sources of heterogeneity [33]. Statistical significance was determined at a *P*<.05. The level of evidence was evaluated using the GRADEpro GDT web-based tool.

## Results

### Study Selection

**Figure 1:**
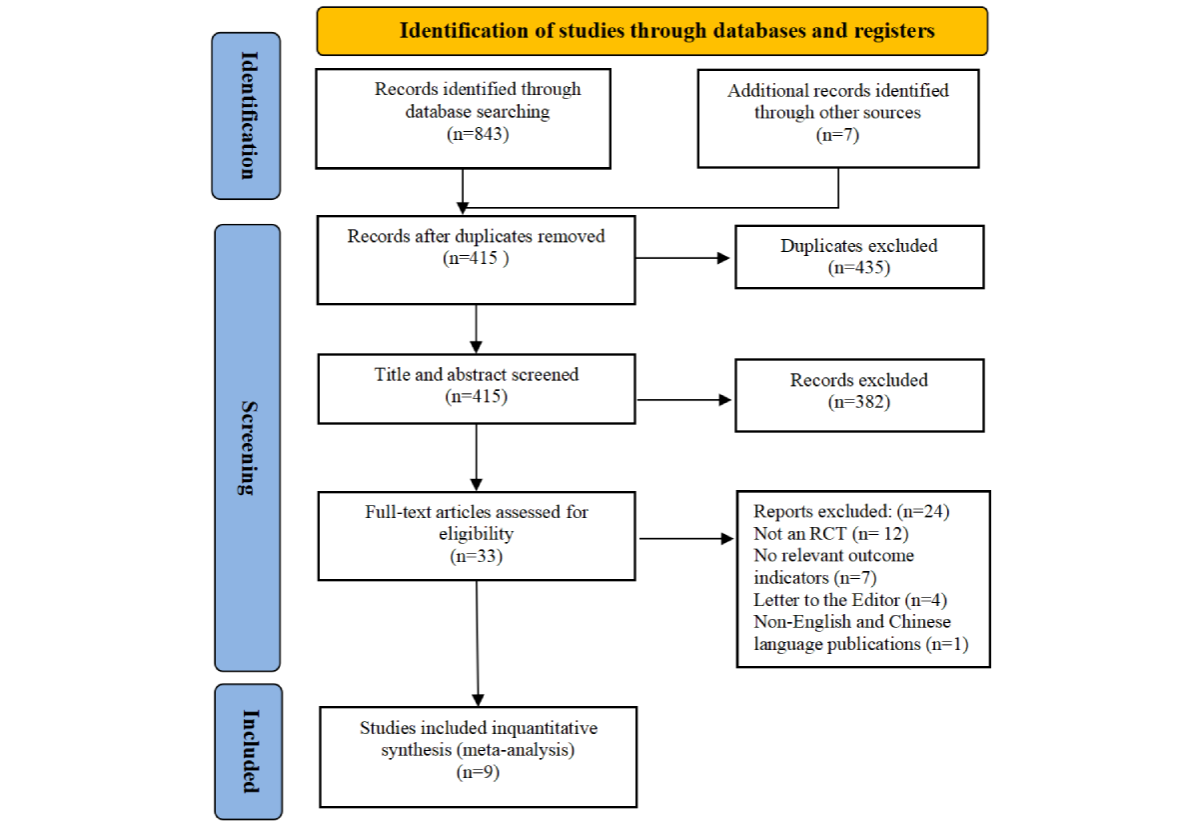
PRISMA (Preferred Reporting Items for Systematic Reviews and Meta-Analyses) flowchart of the selection process. RCT: randomized controlled trial.

After conducting a comprehensive search across various databases, a total of 843 RCTs were found. Additionally, 7 more studies were obtained by snowballing. Following the removal of duplicates, 415 articles were screened based on their titles and abstracts. Out of these, 382 articles were excluded, and the remaining 33 articles were examined in their entirety. Ultimately, a total of 9 full-text articles were considered for quantitative synthesis. This included 5 papers in English and 4 papers in Chinese. More specific information can be found in the study’s PRISMA flowchart (Figure 1).

### Characteristics of Included Studies

We included 9 RCTs [34-42] from 6 countries. There were no statistical differences in general information between the trial and control groups in each study. A total of 791 study participants were included, with 395 in the experimental group taught CPR training using serious games and 396 in the control group taught CPR training using traditional methods. Additional information can be found in Multimedia Appendix 2 [34-42].

### Quality Assessment

**Figure 2:**
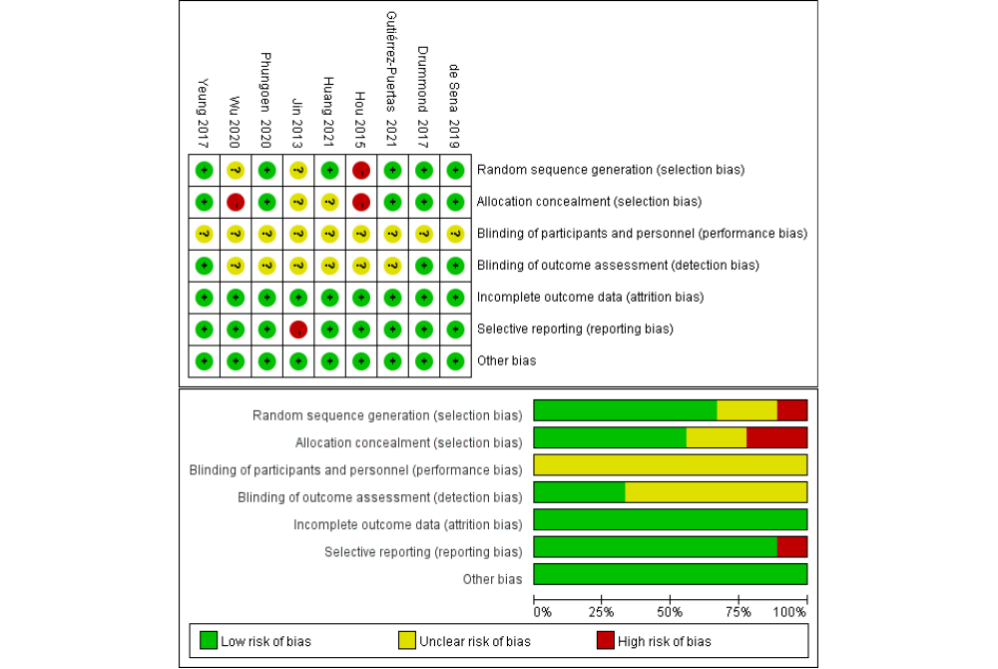
Methodological quality assessment of risk of bias for the included trials.

The risk of bias evaluation of the included literature is presented in Figure 2 [34-42] (the colors green, yellow, and red in the figure mean “low risk of bias,” “unclear risk of bias,” and “high risk of bias,” respectively). The quality of the included studies was found to be acceptable. In 6 RCTs [36-38,40-42], they described the generation of random sequences, of which 5 RCTs [36-38,40,41] described methods of allocation concealment. Due to CPR training and teaching, it was not possible to blind participants. In 3 RCTs [36-38], they applied the blinding method for researchers. Additionally, in 1 RCT [34], they had a high risk of reporting bias, and all 9 RCTs had complete data and did not have any other bias.

### Meta-Analysis Results

#### The Effect of Serious Games Teaching on CPR Theory Performance

**Figure 3:**
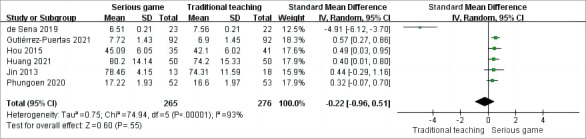
Meta-analysis of the effect of serious games on theory assessment.

In the analysis, 6 out of the 9 studies [34,35,38,40-42] used posttraining CPR theory assessment as an outcome measure in RCTs. The pooled results revealed significant heterogeneity among the studies (*P*<.001; *I*^2^=93%), necessitating the use of a random-effects model for the meta-analysis. Figure 3 [34,35,38,40-42] demonstrates that there was no significant disparity in the theory assessment between the 2 groups under investigation (SMD –0.22, 95% CI –0.96 to 0.51; *P*=.55).

#### The Effect of Serious Games Teaching on the Performance of CPR Skills Operations

**Figure 4:**
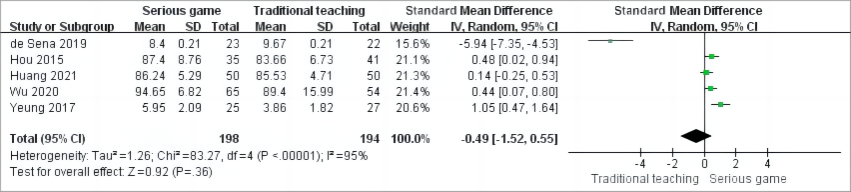
Meta-analysis of the effect of serious games on skill assessment.

Posttraining CPR skill manipulation performance was assessed as an outcome indicator in 5 RCTs [35,36,38,39,42] out of the 9 studies included. Meta-analysis was conducted using a random-effects model due to heterogeneity among the studies (*P*<.001; *I*^2^=95%). The results indicated that there was no significant difference in skills assessment between the 2 study groups (SMD –0.49, 95% CI –1.52 to 0.55; *P*=.36). This suggests that the use of serious games for CPR training did not lead to a significantly different skill level compared to other traditional training methods (Figure 4 [35,36,38,39,42]).

#### The Effect of Serious Games Teaching on the Depth of CPR Compression

**Figure 5:**
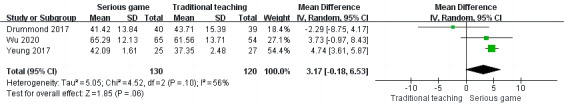
Meta-analysis of the effect of serious games on cardiopulmonary resuscitation (CPR) compression depth.

A total of 3 studies [36,37,39] presented findings on the impact of serious games on CPR compression depth. The assessment of heterogeneity demonstrated variability among the included studies (*P*=.10; *I*^2^=56%), necessitating the application of a random effects model. The analysis depicted in Figure 5 [36,37,39] revealed that the disparity between the 2 groups did not reach statistical significance (MD 3.17, 95% CI –0.18 to 6.53; *P*=.06).

#### The Effect of Serious Games Teaching on the Frequency of CPR Compression

**Figure 6:**
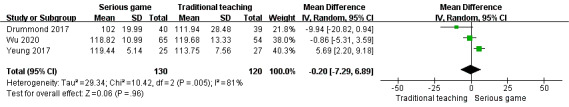
Meta-analysis of the effect of serious games on cardiopulmonary resuscitation (CPR) compression frequency.

A meta-analysis was performed on 3 studies [36,37,39] that investigated the impact of serious games training on the frequency of CPR compression. Due to the variation among these studies (*P*=.005; *I*^2^=81%), a random effects model was used. The results, as illustrated in Figure 6 [36,37,39], indicated that there was no significant difference in the theory of CPR compression rate between the 2 study groups (MD –0.20, 95% CI –7.29 to 6.89; *P*=.96).

### Sensitivity Analysis

**Figure 7:**
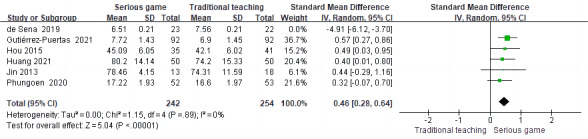
Sensitivity analysis of meta-analysis of cardiopulmonary resuscitation (CPR) theory performance.

**Figure 8:**
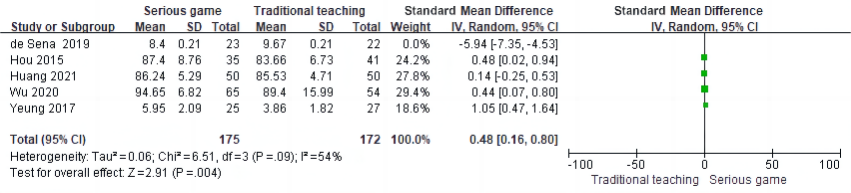
Sensitivity analysis of meta-analysis of cardiopulmonary resuscitation (CPR) skills operations.

**Figure 9:**
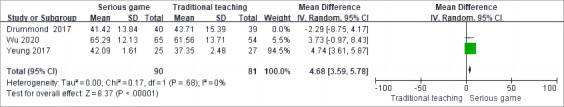
Sensitivity analysis of meta-analysis of cardiopulmonary resuscitation (CPR) compression depth.

**Figure 10:**
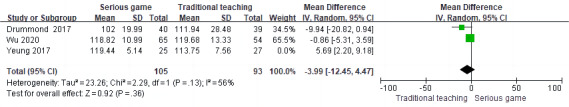
Sensitivity analysis of meta-analysis of cardiopulmonary resuscitation (CPR) compression frequency.

We conducted separate analyses using both fixed effects and random effects models to examine the SMD, MD, and 95% CI of each model. By systematically excluding studies one by one, when the study by de Sena et al [38] was excluded, we observed a decrease in heterogeneity from 93% to 0% for theoretical assessment (Figure 7 [34,35,40-42]) and from 95% to 54% for skill assessment (Figure 8 [35,36,39,42]), respectively. This indicates that the study conducted by de Sena et al [38] may have contributed to the observed heterogeneity. In the meta-analysis of CPR compression depth, heterogeneity decreased from 56% to 0% after the exclusion of the study by Drummond et al [37], indicating that this study was the source of heterogeneity (Figure 9 [36,39]). After the exclusion of the study by Yeung et al [36], the heterogeneity of the meta-analysis on compression frequency of CPR decreased from 81% to 56%, indicating that this study was one of the sources of heterogeneity (Figure 10 [37,39]).

### GRADE Evidence Quality Levels

Table S1 in Multimedia Appendix 3 presents the GRADE (Grading of Recommendations, Assessment, Development, and Evaluations) system evidence level for each outcome indicator in the meta-analysis of this study. The 4 outcome indicators considered were theory assessment, skill assessment, compression depth, and compression frequency.

## Discussion

### Principal Findings

This study systematically evaluated the efficacy of serious games-based training in CPR education, drawing upon data from 9 studies with a total of 791 participants. Our findings reveal no significant differences in theoretical exam scores, skill assessment scores, compression depth, or compression frequency between serious games-based and traditional CPR training methods. This suggests that serious games offer a highly effective alternative for CPR education. In alignment with the 2020 American Heart Association Guidelines for Cardiopulmonary Resuscitation and Emergency Cardiovascular Care [27], which recommend the incorporation of serious games into CPR education, our results underscore the positive impact of virtualized, gamified learning models on knowledge acquisition and CPR skill mastery. Theory and skills assessments are pivotal components of CPR training, serving as key indicators of training effectiveness and student proficiency. Our meta-analysis demonstrates that serious games-based CPR training is on par with traditional methods in enhancing both knowledge acquisition and skill levels. Consequently, serious games represent a valuable addition to the spectrum of CPR teaching and training methods, fostering innovation and aligning with the American Heart Association’s guidelines for modernizing teaching tools and approaches.

### Comparison With Previous Work

This study aligns with a previous meta-analysis [43], indicating that both lay and medical school students exhibit enhanced knowledge following web-based digital resuscitation training. Moreover, they demonstrate comparable cognitive outcomes to those undergoing traditional training sessions. The inclination of younger individuals toward serious games for acquiring new skills stems from their immersive and interactive nature, offering a secure trial-and-error environment [44]. This, coupled with engaging and positive learning experiences, reduces reliance on educational resources and fosters active, independent learning—especially when conventional training methods are inaccessible. This approach helps in sustaining knowledge levels, preventing decay over time, and attaining learning outcomes equivalent to traditional education forms [45]. Despite these benefits, serious games’ applications for CPR training face challenges, presenting a mixed landscape concerning usability and enjoyment quality. Issues range from outdated guidelines and unupdated advice to overly detailed, professional information hindering learning efficacy. Such drawbacks may discourage public engagement with CPR learning [46]. For nonmedical learners, serious games must ensure acceptable usability, simplifying the comprehension and retention of CPR theoretical knowledge. Regular updates aligning with the latest guidelines can transform serious games into dynamic electronic textbooks [46,47]. To maximize the potential of serious games over traditional training, it is crucial to identify and evaluate functions that motivate learners to increase frequency and actively embrace knowledge updates. This strategic approach could position serious games as a superior alternative for enhancing the theoretical understanding of CPR, offering distinct advantages over traditional training methods [46,48].

Numerous guidelines [27,49-51] underscore the primary goal of CPR training: imparting participants with the skills necessary for high-quality CPR. This involves maintaining the correct compression rate and depth, ensuring thoracic recoil, and minimizing interruptions and hyperventilation. However, traditional training methods have presented challenges, particularly for nonmedical personnel [52,53], in mastering these vital competencies. Previous studies [52,54] have noted that simulated scenarios and repetitive practice often fall short of achieving adequate compression depth and frequency. Aksoy [55] and Siqueira et al [56] propose that a CPR teaching mode based on serious games could enhance learners’ motivation and attitude, consequently improving compression quality. This study echoes Lau et al’s [57] systematic review, indicating equivalence between serious games and traditional training methods in enhancing compression depth and frequency. However, electronic CPR training, including serious games, may not independently enhance skills without some influence from instructors, particularly for beginners. In other words, teacher involvement remains crucial to refining CPR skill performance through serious games training. Lim et al [25] discovered that content learned in serious games may not seamlessly transfer to skill operations during assessments, particularly for students with autonomous learning based on serious games. Scores in the pressing position, crucial for CPR quality, were notably worse than those in traditional training. Factors such as incorrect anatomical positions directly impact compression quality, making it challenging to achieve better performance in practical measures such as compression depth and frequency. While there was no significant difference in CPR compression skill or rate between the 2 training models, serious games-based CPR training revealed imperfections. To address this, integrating and emphasizing the impactful elements and advantageous attributes of traditional training into serious games may compensate for their shortcomings in skill practice. This approach has the potential to amplify the comparative advantages of serious games in CPR training.

In summary, the results of this study are similar to those of similar previous systematic reviews or studies. Nevertheless, given the limited number of studies included in this meta-analysis and the low GRADE evidence level, these results warrant cautious interpretation. Therefore, we recommend future CPR training efforts prioritize conducting high-quality, large-sample studies. This will enable a more comprehensive analysis of the effectiveness of serious games-based training, providing substantial evidence for the refinement of guidelines and the development of related teaching methodologies.

### Strengths and Limitations

#### Strengths

This review compensates for the shortcomings of the previous literature in English by focusing on all types of serious games and conducting a comprehensive search of massive Chinese databases. Certainly, this study was conducted in strict accordance with highly recommended guidelines (ie, PRISMA), with early registration of the protocol for the systematic review and final grading of the evidence based on the GRADEpro GDT web-based tool, so it can be considered a robust, high-quality review. In addition, the meta-analysis conducted in this study involved 9 RCTs [34-42]. These RCTs provided detailed information on the study population, training protocol, serious games used, and measurement tools for outcome indicators. As for blinding implementation, it was challenging to blind interventionists due to the nature of CPR teaching training, which resulted in some degree of implementation bias. On the other hand, blinding the measurer effectively prevented measurement bias, particularly when assessing CPR theoretical knowledge and skills. Objective outcome indicators such as CPR compression depth and frequency, as recorded by the simulator, were less susceptible to measurement bias. The literature also addressed missed visits, had a low risk of selective reporting bias, and demonstrated baseline comparability between groups. Therefore, the included literature was of high quality, and the findings can be considered credible.

#### Limitations

This study acknowledges several limitations that merit consideration. First, our research only encompassed studies available in Chinese and English, which may introduce a linguistic bias. Second, heterogeneity in our meta-analysis results emerged due to variations in study populations, the use of different serious games, and diverse tools used to measure outcome indicators. Despite our efforts to explore the sources of heterogeneity through sensitivity analysis, a complete explanation remained elusive. In particular, it is worth noting that the use of different instruments by the included studies to evaluate training outcomes may have influenced the judgment of the results. Third, the relatively small number of included studies prevented us from conducting tests for publication bias. Additionally, some data underwent statistical transformations during the meta-analysis, potentially influencing the accuracy of the results. Lastly, this study focused primarily on CPR theory assessment, skill evaluation, compression depth, and compression rate as outcome indicators, without delving into knowledge and skill retention post-training, trainees’ self-efficacy, or other facets of compression quality.

### Implications for Future Research and Practice

Serious games, as an innovative model for CPR teaching and training, offer a promising avenue for first aid education, catering to diverse populations. However, this approach is still in its developmental and exploratory phases, and its cost-effectiveness warrants discussion. Future research should consider incorporating outcome indicators from the field of health economics to address economic barriers and promote the adoption of serious games in professional medical education and broader first aid training. Additionally, many studies lack standardized training specifications for serious games, including training duration, frequency, trainer intervention levels, and evaluation methods and tools for assessing training effectiveness. While serious games are recommended for CPR education, the specific details of this training mode require further standardization. Moreover, the quality of serious games, which serve as the platform for CPR training, significantly impacts training effectiveness. Developing serious games that align with international guidelines and cater to the diverse characteristics of trainees is undoubtedly challenging but essential. In conclusion, future research should prioritize conducting multicenter, large-sample RCTs to advance our understanding of the potential of serious games in CPR education.

### Conclusion

This study conducted a meta-analysis of RCTs to assess the efficacy of serious games in CPR training. The findings indicate that serious games are equally effective as traditional training methods in enhancing CPR theory assessment and skill evaluation. Meanwhile, no significant differences emerged between serious games and traditional training methods regarding CPR compression depth and frequency. Notably, the current body of high-quality studies on serious games in CPR training is limited, often characterized by small sample sizes. Therefore, future research should prioritize conducting additional high-quality RCTs to provide further evidence and offer a more comprehensive understanding of the impact of serious games in CPR training and education.

## Appendix

Multimedia Appendix 1 Search strategy.

Multimedia Appendix 2 Detailed characteristics of the included trials.

Multimedia Appendix 3 Supplementary table S1.

Multimedia Appendix 4 PRISMA (Preferred Reporting Items for Systematic Reviews and Meta-Analyses) checklist.

## Abbreviations

AED: automated external defibrillator
CPR: cardiopulmonary resuscitation
EMS: emergency medical services
FR: first responder
MD: mean difference
OHCA: out-of-hospital cardiac arrest
PRISMA: Preferred Reporting Items for Systematic Reviews and Meta-Analyses
PROSPERO: International Prospective Register of Systematic Reviews
RCT: randomized controlled trial
SMD: standardized mean difference

## Data Availability

Data sharing is not applicable to this article as no data sets were generated or analyzed during this study.
