# Supplementary material for: The Effects of Serious Games on Cardiopulmonary Resuscitation Training and Education: Systematic Review With Meta-Analysis of Randomized Controlled Trials
Source: JMIR Serious Games. 2024 Feb 6;12:e52990. doi: 10.2196/52990 (PMC10879970; doi:10.2196/52990)
Supplement: Multimedia Appendix 1 [file games_v12i1e52990_app1.docx]

**Multimedia Appendix 1**

Search strategy .

| PubMed | Search string | Results |
| --- | --- | --- |
| #1 | "cardiopulmonary resuscitation"[MeSH Terms] | 22606 |
| #2 | “CPR”[Title/Abstract] | 16160 |
| #3 | “basic life support”[Title/Abstract] | 2545 |
| #4 | “BLS”[Title/Abstract] | 2659 |
| #5 | “first aid training”[Title/Abstract] | 513 |
| #6 | “resuscitation education”[Title/Abstract] | 113 |
| #7 | “emergency skill teaching”[Title/Abstract] | 7 |
| #8 | #1 OR #2 OR #3 OR #4 OR #5 OR #6 OR #7 | 34224 |
| #9 | "education"[Title/Abstract] | 630808 |
| #10 | "training"[Title/Abstract] | 570086 |
| #11 | "learn*"[Title/Abstract] | 637164 |
| #12 | "teach*"[Title/Abstract] | 239545 |
| #13 | "Skill-building"[Title/Abstract] | 949 |
| #14 | #9 OR #10 OR #11 OR #12 OR #13 OR #13 | 1703797 |
| #15 | "serious game"[Title/Abstract] | 801 |
| #16 | "educational game"[Title/Abstract] | 161 |
| #17 | "learning game"[Title/Abstract] | 55 |
| #18 | "gam*"[Title/Abstract] | 2237 |
| #19 | #15 OR #16 OR #17 OR #18 | 3234 |
| #20 | #8 AND #14 AND #19 | 17 |
| #21 | #20 AND (english[Filter]) | 17 |

History and Search Details（Download from pubmed）

| Search number | Query | Filters | Search Details | Results |
| --- | --- | --- | --- | --- |
| 21 | ((((((((cardiopulmonary resuscitation[MeSH Terms]) OR (CPR[Title/Abstract])) OR (basic life support[Title/Abstract])) OR (BLS[Title/Abstract])) OR (first aid training[Title/Abstract])) OR (resuscitation education[Title/Abstract])) OR (emergency skill teaching[Title/Abstract])) AND (((((education[Title/Abstract]) OR (training[Title/Abstract])) OR (learn*[Title/Abstract])) OR (Teach*[Title/Abstract])) OR (Skill-building[Title/Abstract]))) AND ((((serious game[Title/Abstract]) OR (educational game[Title/Abstract])) OR (learning game[Title/Abstract])) OR (gam*[Title/Abstract])) | English | (("cardiopulmonary resuscitation"[MeSH Terms] OR "CPR"[Title/Abstract] OR "basic life support"[Title/Abstract] OR "BLS"[Title/Abstract] OR "first aid training"[Title/Abstract] OR "resuscitation education"[Title/Abstract] OR (("emerge"[All Fields] OR "emerged"[All Fields] OR "emergence"[All Fields] OR "emergences"[All Fields] OR "emergencies"[MeSH Terms] OR "emergencies"[All Fields] OR "emergency"[All Fields] OR "emergent"[All Fields] OR "emergently"[All Fields] OR "emergents"[All Fields] OR "emerges"[All Fields] OR "emerging"[All Fields]) AND "skill teaching"[Title/Abstract])) AND ("education"[Title/Abstract] OR "training"[Title/Abstract] OR "learn*"[Title/Abstract] OR "teach*"[Title/Abstract] OR "Skill-building"[Title/Abstract]) AND ("serious game"[Title/Abstract] OR "educational game"[Title/Abstract] OR "learning game"[Title/Abstract] OR "gam"[Title/Abstract])) AND (english[Filter]) | 17 |
| 20 | ((((((((cardiopulmonary resuscitation[MeSH Terms]) OR (CPR[Title/Abstract])) OR (basic life support[Title/Abstract])) OR (BLS[Title/Abstract])) OR (first aid training[Title/Abstract])) OR (resuscitation education[Title/Abstract])) OR (emergency skill teaching[Title/Abstract])) AND (((((education[Title/Abstract]) OR (training[Title/Abstract])) OR (learn*[Title/Abstract])) OR (Teach*[Title/Abstract])) OR (Skill-building[Title/Abstract]))) AND ((((serious game[Title/Abstract]) OR (educational game[Title/Abstract])) OR (learning game[Title/Abstract])) OR (gam*[Title/Abstract])) |  | ("cardiopulmonary resuscitation"[MeSH Terms] OR "CPR"[Title/Abstract] OR "basic life support"[Title/Abstract] OR "BLS"[Title/Abstract] OR "first aid training"[Title/Abstract] OR "resuscitation education"[Title/Abstract] OR (("emerge"[All Fields] OR "emerged"[All Fields] OR "emergence"[All Fields] OR "emergences"[All Fields] OR "emergencies"[MeSH Terms] OR "emergencies"[All Fields] OR "emergency"[All Fields] OR "emergent"[All Fields] OR "emergently"[All Fields] OR "emergents"[All Fields] OR "emerges"[All Fields] OR "emerging"[All Fields]) AND "skill teaching"[Title/Abstract])) AND ("education"[Title/Abstract] OR "training"[Title/Abstract] OR "learn*"[Title/Abstract] OR "teach*"[Title/Abstract] OR "Skill-building"[Title/Abstract]) AND ("serious game"[Title/Abstract] OR "educational game"[Title/Abstract] OR "learning game"[Title/Abstract] OR "gam"[Title/Abstract]) | 17 |
| 19 | (((serious game[Title/Abstract]) OR (educational game[Title/Abstract])) OR (learning game[Title/Abstract])) OR (gam*[Title/Abstract]) |  | "serious game"[Title/Abstract] OR "educational game"[Title/Abstract] OR "learning game"[Title/Abstract] OR "gam"[Title/Abstract] | 3,234 |
| 18 | gam*[Title/Abstract] |  | "gam"[Title/Abstract] | 2,237 |
| 17 | learning game[Title/Abstract] |  | "learning game"[Title/Abstract] | 55 |
| 16 | educational game[Title/Abstract] |  | "educational game"[Title/Abstract] | 161 |
| 15 | serious game[Title/Abstract] |  | "serious game"[Title/Abstract] | 801 |
| 14 | ((((education[Title/Abstract]) OR (training[Title/Abstract])) OR (learn*[Title/Abstract])) OR (Teach*[Title/Abstract])) OR (Skill-building[Title/Abstract]) |  | "education"[Title/Abstract] OR "training"[Title/Abstract] OR "learn*"[Title/Abstract] OR "teach*"[Title/Abstract] OR "Skill-building"[Title/Abstract] | 1,703,797 |
| 13 | Skill-building[Title/Abstract] |  | "Skill-building"[Title/Abstract] | 949 |
| 12 | Teach*[Title/Abstract] |  | "teach*"[Title/Abstract] | 239,545 |
| 11 | learn*[Title/Abstract] |  | "learn*"[Title/Abstract] | 637,164 |
| 10 | training[Title/Abstract] |  | "training"[Title/Abstract] | 570,086 |
| 9 | education[Title/Abstract] |  | "education"[Title/Abstract] | 630,808 |
| 8 | ((((((cardiopulmonary resuscitation[MeSH Terms]) OR (CPR[Title/Abstract])) OR (basic life support[Title/Abstract])) OR (BLS[Title/Abstract])) OR (first aid training[Title/Abstract])) OR (resuscitation education[Title/Abstract])) OR (emergency skill teaching[Title/Abstract]) |  | "cardiopulmonary resuscitation"[MeSH Terms] OR "CPR"[Title/Abstract] OR "basic life support"[Title/Abstract] OR "BLS"[Title/Abstract] OR "first aid training"[Title/Abstract] OR "resuscitation education"[Title/Abstract] OR (("emerge"[All Fields] OR "emerged"[All Fields] OR "emergence"[All Fields] OR "emergences"[All Fields] OR "emergencies"[MeSH Terms] OR "emergencies"[All Fields] OR "emergency"[All Fields] OR "emergent"[All Fields] OR "emergently"[All Fields] OR "emergents"[All Fields] OR "emerges"[All Fields] OR "emerging"[All Fields]) AND "skill teaching"[Title/Abstract]) | 34,224 |
| 7 | emergency skill teaching[Title/Abstract] |  | ("emerge"[All Fields] OR "emerged"[All Fields] OR "emergence"[All Fields] OR "emergences"[All Fields] OR "emergencies"[MeSH Terms] OR "emergencies"[All Fields] OR "emergency"[All Fields] OR "emergent"[All Fields] OR "emergently"[All Fields] OR "emergents"[All Fields] OR "emerges"[All Fields] OR "emerging"[All Fields]) AND "skill teaching"[Title/Abstract] | 7 |
| 6 | resuscitation education[Title/Abstract] |  | "resuscitation education"[Title/Abstract] | 113 |
| 5 | first aid training[Title/Abstract] |  | "first aid training"[Title/Abstract] | 513 |
| 4 | BLS[Title/Abstract] |  | "BLS"[Title/Abstract] | 2,659 |
| 3 | basic life support[Title/Abstract] |  | "basic life support"[Title/Abstract] | 2,545 |
| 2 | CPR[Title/Abstract] |  | "CPR"[Title/Abstract] | 16,160 |
| 1 | cardiopulmonary resuscitation[MeSH Terms] |  | "cardiopulmonary resuscitation"[MeSH Terms] | 22,606 |
