# Supplementary material for: The Effects of Serious Games on Cardiopulmonary Resuscitation Training and Education: Systematic Review With Meta-Analysis of Randomized Controlled Trials
Source: JMIR Serious Games. 2024 Feb 6;12:e52990. doi: 10.2196/52990 (PMC10879970; doi:10.2196/52990)
Supplement: Multimedia Appendix 2 [file games_v12i1e52990_app2.docx]

**Multimedia Appendix 2.**

Detailed characteristics of the included trials^a-d^.

| Author [reference];Country | Participants | Game Name | Description | | Overall findings | Tools |
| --- | --- | --- | --- | --- | --- | --- |
|  |  |  | Experimental group | Control group |  |  |
| Jin et al[34];China | Medical Students | MicroSim | Through the multimedia room computer physician training software MicroSim learn CPR^a^ related knowledge points and defibrillation operation points. During this period, students can study the content of the material on their own. | Students learn CPR knowledge and skill points on their own by watching AHA^b^ instructional CD^c^ and instructional videos, and practice their skills freely on the simulator, during which students can study the materials on their own. | Theory assessment;others | AHA Basic Life Support and Advanced Life Support Training Examination Paper |
| Hou et al[35];China | Non-medical undergraduates | Not clear | Multimedia courseware teaching, CPR simulator operation and educational game software are used to teach. After that, students practiced on site for 30min, and teachers were responsible for guidance and Q&A^d^. | Using multimedia courseware teaching and CPR simulator operation, students practice on-site operation for 30min, teachers are responsible for guidance and Q&A. | Theory assessment;skill assessment;others | Self-developed measurement tools |
| Yeung et al[36];United Kingdom | Middle school students | Lifesaver | Students learn CPR first aid on tablets using Lifesaver without professional instructor guidance and practice. | CPR training course with face-to-face instruction by instructors. Students take turns practicing CPR skills on mannequins. Instructor to student ratio of 1:6. | Skill assessment;compression depth;compression frequency | Self-developed measurement tools, simulator equipment with data feedback |
| Drummond et al[37];France | Medical Students | Staying alive | Learn the same content through Staying alive game. | Online class to learn CPR knowledge points. | Compression depth;compression frequency | Simulator device with data feedback |
| de Sena et al[38];Brazil | Medical Students | Not clear | Learning the same content through serious games. | Online class to learn CPR knowledge points. | Theory assessment;skill assessment;others | Self-developed measurement tools |
| Wu et al[39];China | High School Students | QCPR Race | The cell phone interactive quiz game was used to learn theoretical knowledge of cardiac first aid, and QCPR classroom simulator was used to practice CPR operation to improve the quality of compressions. | Chest compressions were practiced using a compression feedback system. | Skill assessment;compression depth;compression frequency;other | QCPR classroom simulation equipment to collect data |
| Phungoen et al[40];Thailand | Medical Students | Resus Days | Learn theoretical knowledge in traditional lecture mode and play Resus Days on your own cell phone during the training. | Traditional lecture mode to learn theoretical knowledge, under the guidance of the instructor to carry out CPR practice. | Theory assessment;others | Self-developed measurement tools |
| Gutiérrez-Puertas et al[41];Spain | Nursing students | Guess it | Learning CPR theory through Guess it game. | Learning CPR theory through traditional classroom lectures. | Theory assessment;others | Self-developed measurement tools |
| Huang et al[42];China | Nursing students | Not clear | Four hours of theory lecture followed by 4 hours of computer CPR game experience course. | Four hours of theory lecture followed by 4 hours of skill practice under the guidance of the instructor. | Theory assessment;skill assessment;other | Self-developed evaluation questionnaire, AHA skills assessment evaluation form |

^a^**CPR:** cardiopulmonary resuscitation.

^b^**AHA:** American Heart Association.

^c^**CD:**compact disc.

^d^**Q&A:**Question and Answer.
