# Supplementary material for: The Effects of Serious Games on Cardiopulmonary Resuscitation Training and Education: Systematic Review With Meta-Analysis of Randomized Controlled Trials
Source: JMIR Serious Games. 2024 Feb 6;12:e52990. doi: 10.2196/52990 (PMC10879970; doi:10.2196/52990)
Supplement: Multimedia Appendix 3 [file games_v12i1e52990_app3.docx]

**Table S1.** Detailed summary of findings using the GRADEpro approach^a-c^.

| Certainty assessment | | | | | | | Effect | Certainty |
| --- | --- | --- | --- | --- | --- | --- | --- | --- |
| Outcome indicators | Study design | Risk of bias | Inconsistency | Indirectness | Accuracy | Other considerations | Absolute（95% CI） |  |
| theory assessment (6) | RCT | not serious | very serious^a^ | not serious | not serious | none | -0.22  （-0.96，0.51） | ⊕⊕〇〇  Low |
| skill assessment (5) | RCT | not serious | very serious^a^ | Serious^b^ | Serious^c^ | none | -0.49  （-1.52，0.55） | ⊕〇〇〇  lower |
| compression depth (3) | RCT | not serious | very serious^a^ | Serious^b^ | not serious | none | 3.17  （-0.18，6.53） | ⊕⊕〇〇  Low |
| compression frequency (3) | RCT | not serious | very serious^a^ | Serious^b^ | not serious | none | -0.20  （-7.29，6.89） | ⊕⊕〇〇  Low |

^a^There is a significant amount of variability among studies, and it is not possible to provide a complete explanation for this.

^b^The study's outcome was assessed through the utilization of an internet-based formula calculator for data conversion.

^c^The sample size of the study is small, and the confidence interval is relatively wide.
